# Underdamped scaled Brownian motion: (non-)existence of the overdamped limit in anomalous diffusion

**DOI:** 10.1038/srep30520

**Published:** 2016-07-27

**Authors:** Anna S. Bodrova, Aleksei V. Chechkin, Andrey G. Cherstvy, Hadiseh Safdari, Igor M. Sokolov, Ralf Metzler

**Affiliations:** 1 Institut für Physik, Humboldt-Universität zu Berlin, Newtonstrasse 15, 12489 Berlin, Germany; 2 Faculty of Physics, M.V.Lomonosov Moscow State University, Moscow, 119991, Russia; 3 Akhiezer Institute for Theoretical Physics, Kharkov Institute of Physics and Technology, Kharkov 61108, Ukraine; 4 Institute of Physics and Astronomy, University of Potsdam, 14476 Potsdam, Germany; 5 Department of Physics & Astronomy, University of Padova, 35122 Padova, Italy; 6 Department of Physics, Shahid Beheshti University, G.C., Evin, Tehran 19839, Iran

## Abstract

It is quite generally assumed that the overdamped Langevin equation provides a quantitative description of the dynamics of a classical Brownian particle in the long time limit. We establish and investigate a paradigm anomalous diffusion process governed by an underdamped Langevin equation with an explicit time dependence of the system temperature and thus the diffusion and damping coefficients. We show that for this underdamped scaled Brownian motion (UDSBM) the overdamped limit fails to describe the long time behaviour of the system and may practically even not exist at all for a certain range of the parameter values. Thus persistent inertial effects play a non-negligible role even at significantly long times. From this study a general questions on the applicability of the overdamped limit to describe the long time motion of an anomalously diffusing particle arises, with profound consequences for the relevance of overdamped anomalous diffusion models. We elucidate our results in view of analytical and simulations results for the anomalous diffusion of particles in free cooling granular gases.

The mean squared displacement (MSD) of a Brownian particle at sufficiently long times follows the linear time dependence 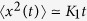, as predicted by the second Fick’s law^1^ and physically explained by Einstein^2^ and Smoluchowski^3^. However, already in 1926 Richardson reported the distinct non-Fickian behaviour of tracer particles in atmospheric turbulence^4^. Today, such *anomalous diffusion* is typically associated with the power-law form


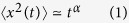


of the MSD, where subdiffusion corresponds to values of the anomalous diffusion exponent *α* in the range 0 < *α* < 1 and superdiffusion to *α* > 1^5^,^6^,^7^,^8^. Classical examples for subdiffusion include the charge carrier motion in amorphous semiconductors^9^, the spreading of tracer chemicals in subsurface aquifers^10^ or in convection rolls^11^, as well as the motion of a tracer particle in a single file of interacting particles^12^. Superdiffusion is known from tracer motion in turbulent flows^4^ and weakly chaotic systems^13^, or for randomly searching, actively moving creatures such as microorganisms and bacteria^14^, albatrosses^15^, or humans^16^.

Modern microscopic techniques, in particular, superresolution microscopy, have led to the discovery of a multitude of anomalous diffusion processes in living biological cells and complex fluids^8^,^17^,^18^,^19^. Thus subdiffusion was observed in live cells for RNA molecules^20^, chromosomal telomeres^21^, or submicron lipid^22^ and insulin granules^23^. Even small proteins such as GFP were demonstrated to subdiffuse^24^. In artificially crowded systems, subdiffusion is also routinely observed^25^,^26^,^27^,^28^. Superdiffusion of injected as well as endogenous submicron particles, due to active processes such as molecular motor driven transport was reported in the cellular context^29^,^30^,^31^. Following the progress of supercomputing capabilities, subdiffusion was also reported for complex molecular systems such as relative diffusion in single proteins^32^, and for constituent molecules in pure^33^,^34^ and crowded^35^,^36^ lipid bilayer membranes^37^.

Apart from the power-law anomalous diffusion (1) ultraslow processes with a logarithmic time dependence


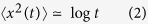


of the MSD exist in a variety of systems^8^. Such logarithmic time dependencies occur in Sinai diffusion in quenched random energy landscapes^38^,^39^, periodically iterated maps^40^, colloidal hard sphere systems at the liquid-glass transition^41^, random walks on bundled structures^42^, or in single file diffusion with power-law trapping time distributions for individual particles^43^. A particular system in which ultraslow diffusion occurs are granular gases in the homogeneous cooling stage, in which each particle-particle collision reduces the kinetic energy of the two particles by a constant factor, the so called restitution coefficient^44^.

The nature of anomalous diffusion of the forms (1) or (2) is non-universal and may originate from numerous physical processes. Power-law anomalous diffusion, for instance, emerges for continuous time random walk processes with scale-free distributions of waiting times or jump lengths^9^,^45^, generalised Langevin equations of fractional Brownian motion with power-law correlated, Gaussian noise input^46^, or diffusion processes with deterministic^47^ or random^48^ position dependence of the diffusivity. Ultraslow diffusion can be described in terms of continuous time random walks with super heavy-tailed waiting times^39^,^49^ or heterogeneous diffusion processes with exponential space dependence of the diffusivity^50^.

The motion of a particle of mass *m* in a thermal bath is typically described by a Langevin equation^51^,^52^. While the short time motion of this particle is ballistic, once collision events become relevant, a crossover to normal Brownian motion with MSD (1) and *α* = 1 occurs. The corresponding crossover time scale is given by the inverse friction coefficient. For Brownian motion at sufficiently long times it is sufficient to use the overdamped Langevin equation without the inertia term, to quantitatively describe the particle motion. In other words, the long time limit of the full Langevin equation including the Newton term 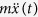 coincides with the solution of the overdamped Langevin equation^52^,^53^.

Here we study a simple anomalous diffusion process based on the full Langevin equation with inertial term and a time dependent diffusion coefficient. For this underdamped scaled Brownian motion (UDSBM) we demonstrate that the long time limit may be distinctly disparate from the analogous overdamped process due to extremely persistent inertial effects, that dominate the particle motion on intermediate-asymptotic time scales. This a priori surprising finding breaks with a commonly accepted dogma for stochastic processes and demonstrates that the correct mathematical description for particles with a mass in the long time limit for anomalous diffusion processes may be a delicate issue, that requires special care. Our findings are based on analytical calculations and confirmed by extensive stochastic simulations. Comparison to event driven simulations of granular gases confirm the results of our UDSBM model for a physical model based on first principles.

To proceed, we first provide a concise summary of the properties of the regular underdamped Langevin equation for Brownian motion and its overdamped limit. The following Section then briefly introduces the overdamped Langevin description for scaled Brownian motion (SBM) corresponding to the UDSBM process without the inertia term. The subsequent section then introduces the full Langevin equation for UDSBM including the mass term. We unravel the ensemble and time averaged characteristics of this UDSBM process analytically and show the agreement with stochastic simulations. Both cases of power-law anomalous diffusion (1) as well as ultraslow diffusion (2) are considered. In particular, we also present a comparison of the UDSBM process with event driven simulations of a cooling granular gas. Mathematical details of the derivations are presented in the Methods section.

## Langevin Equation with Constant Coefficients

In this section we briefly recall the basic properties of the stochastic description of Brownian motion, in particular, the transitions from the under- to the overdamped regimes. We consider both the more traditional ensemble averages of moments and the corresponding time averages, important for the analysis of time series obtained from particle tracking experiment and simulations^8^,^18^.

### Overdamped Langevin equation

Let us start with the overdamped Langevin equation with the constant diffusion coefficient *D*_0_^52^,^53^,


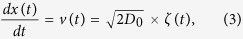


fuelled by the Gaussian noise *ζ*(*t*) with *δ*-correlation


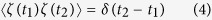


and zero mean 〈*ζ*(*t*)〉 = 0. The corresponding MSD has the linear time dependence


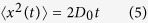


expected for overdamped Brownian motion of a test particle in a thermal bath. The noise strength is given by the diffusion constant *D*_0_.

In the single particle tracking experiments and massive computer simulations often only few but long traces are available for the analysis. In this case one typically analyses the particle motion encoded in the time series *x*(*t*) via the time averaged MSD^8^,^18^


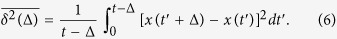


Here Δ is the lag time and *t* denotes the total length of the trajectory (measurement time). An additional average over *N* time traces *x*_*i*_(*t*)


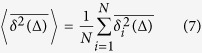


then produces a smooth variation of the time averaged MSD with the lag time. For Brownian motion we observe the equality 〈[*x*(*t*′ + Δ) − *x*(*t*′)]^2^〉 ~ 〈*δx*^2^〉 × Δ/*τ*, where 〈*δx*^2^〉 is the variance of the underlying jump length distribution, and *τ* is the typical time for a single jump^8^,^18^. We therefore obtain the equality


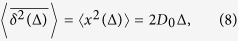


so that the system is ergodic in the Boltzmann-Khinchin sense, that is, time and ensemble averages coincide. In particular, we see that the time averaged MSD 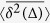 is independent of the observation time *t*, reflecting the stationarity of the process.

### Underdamped Langevin equation

Now consider the underdamped Langevin equation with inertial term^52^,^53^,


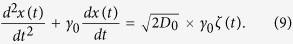


The constant damping coefficient *γ*_0_ and the diffusion coefficient *D*_0_ are connected via the Einstein-Smoluchowski-Sutherland fluctuation dissipation relation


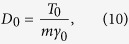


where we use the convention to set the Boltzmann constant *k*_*B*_ to unity. The two point velocity correlation function encoded by the underdamped Langevin equation (9) decays exponentially in the time difference,


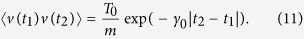


The associated characteristic time is defined by the inverse of the friction coefficient, 1/*γ*_0_. The MSD follows from the velocity correlation function via


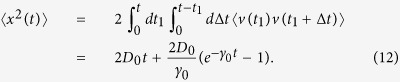


At short times *t* ≪ 1/*γ*_0_ the MSD scales ballistically, 
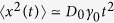
 while at long times *t* ≫ 1/*γ*_0_ the MSD is given by the linear time dependence (5) of the overdamped Langevin equation. Thus the inertial effects indeed cancel out rapidly and are important only at times smaller than or comparable to the characteristic time scale 1/*γ*_0_.

For the underdamped Langevin equation the time averaged MSD is calculated using Eqs (6) and (7). It has the same time dependence as the ensemble averaged MSD, namely,


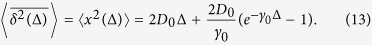


In addition to this ergodic behaviour, we have thus corroborated that the dynamic encoded in the overdamped Langevin equation (3) exactly equals the long time limit of the underdamped Langevin equation (9).

## Scaled Brownian Motion

Scaled Brownian motion (SBM) designates an anomalous diffusion process based on an overdamped Langevin equation fuelled by white Gaussian noise, see below. SBM involves a power law time dependent diffusion coefficient 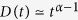 54,55,56,57,58, stemming from a time dependence of the system temperature, see below. SBM is a quite simple process, as it is Markovian. Concurrently, it is strongly non-stationary. For this reason the process stays time dependent even in a confining external potential and is weakly non-ergodic as well as ageing in the sense defined below^55^,^56^,^57^,^58^.

SBM should not be confused with fractional Langevin equation motion or fractional Brownian motion which are non-Markovian yet Gaussian processes with stationary increments whose probability density in the overdamped limit coincides with that of SBM but have a completely different physical origin^8^,^46^,^59^. The underdamped Langevin equation for fractional Langevin equation motion was analysed in refs 27 and 60, 61, 62 and shown to exhibit interesting effects such as oscillatory behaviour of the velocity correlations as well as transient ageing and non-ergodic behaviour. However, these decay rather quickly to make way for the expected overdamped behaviour. Here we show that the behaviour of UDSBM is significantly different from the fractional Langevin equation motion and involves persistent inertial terms.

Before starting the discussion of SBM we note that anomalous diffusion with time dependent diffusion coefficient 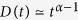 occurs, for instance, in the famed Batchelor model for turbulent diffusion^63^. SBM was used to model the water diffusion in brain measured by magnetic resonance imaging^64^, the mobility of proteins in cell membranes^65^, or the motion of molecules in porous environments^66^. As effective subdiffusion model it was also used to describe biological systems^67^,^68^,^69^. Physically time dependent diffusion coefficients arise naturally in systems with a time dependent temperature such as melting snow^70^,^71^ or free cooling granular gases, in which the temperature is given by the kinetic energy, which dissipates progressively into internal degrees of freedom of the gas particles^44^,^72^,^73^.

### Scaled Brownian motion with *α* > 0

The overdamped SBM Langevin equation with time dependent diffusion coefficient 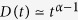 and *α* > 0 is typically used as the definition of SBM^54^,^55^,^56^,^57^,^58^,


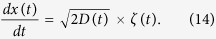


Here we consider the time dependent diffusion coefficient in the more general form


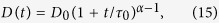


which avoids a singular behaviour at *t* = 0, and *τ*_0_ represents a characteristic time for the mobility variation. For this choice *D*_0_ = *D*(0) is the initial diffusion coefficient. The specific form (15) of *D*(*t*) is primarily motivated by the corresponding expression derived in the theory of cooling granular gases^74^. In addition Eq. (15) represents a simple smooth function allowing us to reproduce all three regimes in the evolution of the MSD we are interested in in what follows, namely, ballistic, normal, and anomalous.

Given definition (15) the mean squared displacement follows in the form


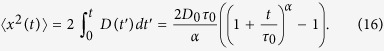


Thus the MSD grows linearly, 〈*x*^2^(*t*)〉 ~ 2*D*_0_*t* at short times *t* ≪ *τ*_0_. At long times *t* ≫ *τ*_0_ it scales according to Eq. (1) and thus covers both sub- and superdiffusive processes^54^,^55^,^56^,^57^,^58^.

The full expression for the time averaged MSD is given by Eq. (42) in the Methods section. At short times Δ ≪ *t* ≪ *τ*_0_ the diffusion coefficient is almost unchanged, *D*(*t*) ≈ *D*_0_ and normal ergodic behaviour is observed, 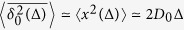. At longer lag times *τ*_0_ ≪ Δ ≪ *t* we get that


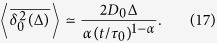


Thus the MSD and the time averaged MSD exhibit a fundamentally different (lag) time dependence, a weak breaking of ergodicity. In contrast to the Langevin equation with constant coefficients the time averaged MSD now also depends on the measurement time *t*, a phenomenon called ageing^8^.

### Ultraslow SBM with *α* = 0

Ultraslow SBM corresponds to the limiting case *α* = 0 for the diffusion coefficient (Equation 15)^75^,


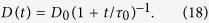


In this case the MSD has the logarithmic time dependence


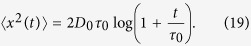


At long times the MSD 〈*x*^2^(*t*)〉 converges to Eq. (2). The full expression for the time averaged MSD is given by Eq. (61) in Methods. For *τ*_0_ ≪ Δ ≪ *t* the time averaged MSD has the following mixed power-law-logarithmic scaling^75^


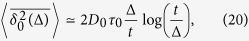


which again features an ageing behaviour^57^,^58^. At short times Δ ≪ *τ*_0_, *t* ≪ *τ*_0_ normal diffusion is observed, 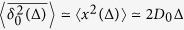.

## Results

### Underdamped scaled Brownian motion

Let us now turn to the UDSBM case and consider the underdamped version of the Langevin equation (14) with time dependent diffusion and damping coefficients, *D*(*t*) and *γ*(*t*), respectively,


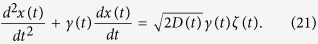


In that sense it is a straightforward extension of the Brownian Langevin equation (9) with additional multiplicative coefficients. We assume that the particle moves in a bath with temperature *T*(*t*) with power law time dependence


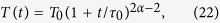


where *α* ≥ 0 and the value *T*_0_ = *T*(0) is the initial temperature. The time scale *τ*_0_ corresponds to the characteristic time of the temperature decay. Larger *τ*_0_ values imply a slower temperature decrease. In the limit *τ*_0_ = ∞ the temperature of the system remains constant, which corresponds to the case of normal diffusion. We assume that the bath is in local equilibrium, and the time dependent damping coefficient scales as 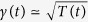 or


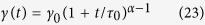


with the initial value *γ*_0_ = *γ*(0). Thus 1/*γ*(*t*) defines the characteristic decay time of the velocity correlation function, which is now also time dependent. The choice of the damping coefficient in the form (23) appears natural since it is in accordance with the two paradigmatic models. The first one corresponds to a massive Brownian particle in a gas with continuous heating or cooling, consisting of elastically colliding particles: in this case the damping coefficient may be derived as a Stokes friction coefficient and is proportional to the dynamical viscosity which in turn scales as 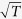
76. The second model corresponds to the self-diffusion in granular gases. In that case the damping coefficient is equal to the inverse velocity autocorrelation time, 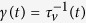, where 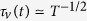 44.

The time dependent diffusion coefficient may then be related to the damping coefficient according to the (time local) fluctuation dissipation theorem^55^,^77^,


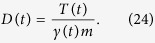


This way we recover the diffusion coefficient (15) introduced above with the initial value *D*_0_ = *T*_0_/(*γ*_0_*m*). In the picture of the cooling granular gas the decrease of the granular temperature due to dissipative collisions of particles according to Eq. (22) was indeed observed^44^. Here the case *α* = 0 considered in subsection B corresponds to particles colliding with constant restitution coefficient^78^, and *α* = 1/6 to granular gases of viscoelastic particles colliding with relative velocity dependent restitution coefficient^44^. The diffusion coefficient in the granular gases decays according to Eq. (15)^44^,^77^,^79^,^80^,^81^,^82^,^83^ and the motion of granular particles slows down continuously while the inter-collision times become longer on average. The underdamped Langevin equation (21) is thus valid for both the description of an underdamped Brownian particle in a bath with time dependent temperature and for the self-diffusion in free cooling granular gases, as will be elaborated further below. The Langevin approach is justified if the typical temperature variation time scale *τ*_0_ is sufficiently larger than the inverse initial damping coefficient, *τ*_0_*γ*_0_ ≫ 1. This time scale separation allows us to introduce the local fluctuation dissipation theorem (24). We stop to note that there is an alternative version of the Langevin equation with time dependent temperature derived for a different system of a Brownian particle interacting with a bath of harmonic oscillators^84^.

Introducing the power-law time dependent diffusion coefficient (15) and damping coefficient (23) into the Langevin equation (21) we obtain


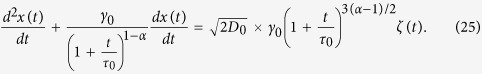


We may expect that the first inertial term in this equation for pronounced subdiffusion (*α* ≪ 1) will behave as *v*/*t* at long times, while the second term scales as *v*/*t*^1−*α*^. For *α* > 1 at long measurement times *t* the overdamped limit always dominates. However, as we will show there exists a long lasting intermediate regime in which the motion of the particles may not be described in terms of the overdamped approximation since both terms have comparable contributions as long as *α* is sufficiently small. This means that particularly for pronounced subdiffusion as in the viscoelastic granular gas with *α* = 1/6 inertial effects play a significant role and thus delay the crossover to the true overdamped limit. In contrast, for superdiffusion this effect is negligible. In the limit of ultraslow underdamped Langevin equation discussed below even for long times both inertial and frictional terms have the same order of magnitude 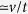, so the underdamped behaviour practically dominates the entire evolution of the system. Such effects will be clarified in detail when we consider the behaviour of MSD and time averaged MSD below.

Before proceeding we note that the bivariate Fokker-Planck equation (Klein-Kramers equation) corresponding to the Langevin equation (25) reads


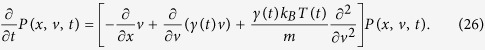


Here *P*(*x*, *v*, *t*) is the probability density function to find the text particle with velocity *v* at time *t*. While this equation could be solved for *P*(*x*, *v*, *t*) after dual Fourier transformation in *x* and *v* as well as Laplace transformation with respect to time *t*, our strategy here is based on the Langevin equation formulation of UDSBM, as the latter allows us to immediately obtain the two-point correlations to calculate the time averaged MSD. We also note that from the formulation (26) we could read off the formal relation 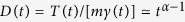 between the time-dependent diffusion coefficient and the time-dependent temperature and friction coefficients, corresponding to the above local fluctuation dissipation relation (24). However, we stress again that UDSBM is an intrinsically non-stationary process off thermal equilibrium^55^.

#### Underdamped scaled Brownian motion with *α* > 0

We first concentrate on the details of the case *α* > 0. Both MSD and time averaged MSD may be derived from the velocity correlation function, which has the following form


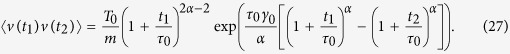


**Figure 1:**
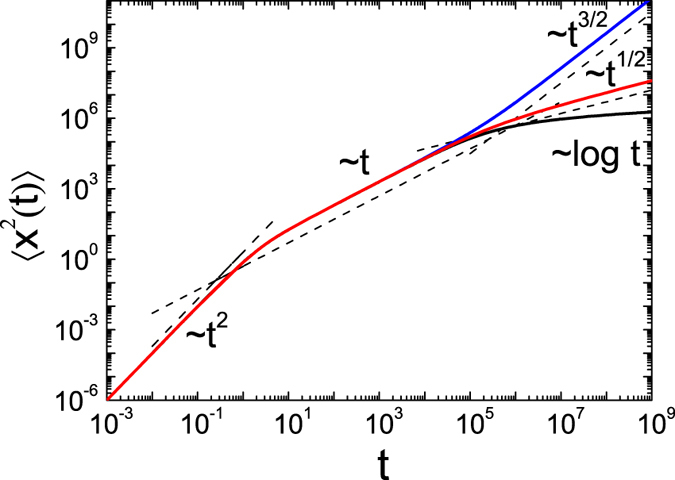
MSD 〈*x*^2^(*t*)〉 according to Eq. (28) for *α* > 0 and Eq. (32) for *α* = 0 for the parameters *τ*_0_ = 100000, *γ*_0_ = 1 with *α* = 3/2 (blue line), *α* = 1/2 (red line), and *α* = 0 (black line). At short times *t* ≪ 1/*γ*_0_ the MSD scales ballistically, 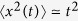, at intermediate times 1/*γ*_0_ ≪ *t* ≪ *τ*_0_ a linear scaling 〈*x*^2^(*t*)〉 ~ *t* is observed, while at long times *t* ≫ 1/*γ*_0_ the asymptotic regime 〈*x*^2^(*t*)〉 ~ *t*^*α*^ is reached for *α* > 0, in the case *α* = 0 we observe 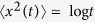.

The full expression for the MSD then reads


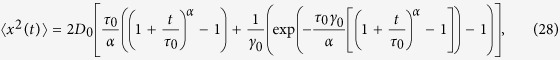


which is valid as long as *τ*_0_*γ*_0_ ≫ 1, which in turn is essential for the validity of our Langevin equation approach. At short times corresponding to 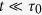 when the temperature has not changed significantly the MSD scales according to Eq. (12). At times *t* ≪ 1/*γ*_0_ compared to the scale set by the damping coefficient the MSD has the ballistic time dependence 〈*x*^2^(*t*)〉 ~ (*T*_0_/*m*)*t*^2^, which cannot be observed for the overdamped version, SBM. At intermediate times 1/*γ*_0_ ≪ *t* ≪ *τ*_0_ the MSD scales according to the normal diffusion law 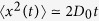. At long times *t* ≫ *τ*_0_ the MSD follows the power-law scaling for overdamped SBM, 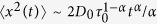. All evolution regimes are depicted in Fig. 1 for *α* = 3/2 (blue line) and *α* = 1/2 (red line). The ultraslow case *α* = 0, shown with the black line, is considered below. It may be seen that at times *t* ≪ *τ*_0_ the behaviour of the MSD is independent of *α* while the *α* dependence becomes apparent at long times.

For the derivation of the time averaged MSD we follow the same approach as described in ref. 74. It may be written as a sum of two terms,


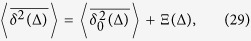


where the first term 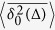 corresponds to the time averaged MSD (17) obtained in the framework of the overdamped equation (14) for SBM. The second term specified in Eq. (43) accounts for the inertial effects. This term is negative and reduces the amplitude of the time averaged MSD as compared to the overdamped case. For short lag times Δ ≪ 1/*γ*_0_ the ballistic regime 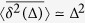 is obtained, as expected. For long lag times 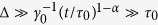 the inertial effects become negligible and the time averaged MSD converges to the time averaged MSD (17) for overdamped SBM. For superdiffusion with *α* > 1 and subdiffusion with values of *α* close to unity the result obtained in the overdamped limit, Eq. (17), holds true for almost the entire range of lag times Δ ≫ *τ*_0_.

This behaviour changes drastically for more pronounced subdiffusion. Namely, we find that for intermediate lag times 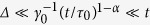 the inertial term Ξ(Δ) becomes comparable to the overdamped term 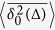, as demonstrated in Methods. The time averaged MSD exhibits an intermediate scaling that is not very distinctive in the case of superdiffusion, and even in the case of subdiffusion as long as *α* is close to unity. A significant correction occurs only for sufficiently small values of *α*, that is, for pronounced subdiffusion. This remarkable appearance of significant corrections, due to persistent ballistic contributions, of the underdamped motion with respect to the overdamped SBM description for subdiffusion is our first main result. It demonstrates that in a simple yet non-stationary process the naive description of a system in terms of the overdamped theory may lead to wrong conclusions. To our knowledge this is the first time that such an observation for diffusive systems is made.

**Figure 2:**
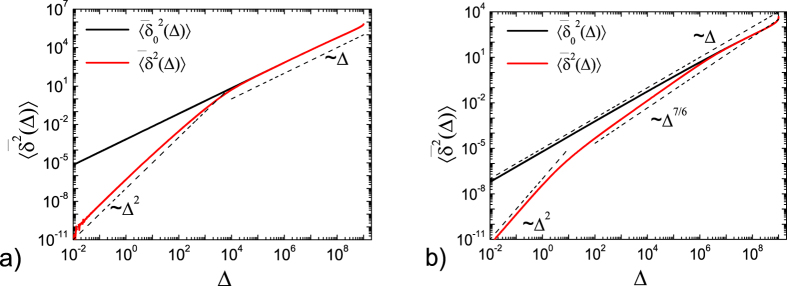
Time averaged MSD in the overdamped limit, 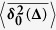 from numerical integration of Eq. (42) (black line) and in the full underdamped case, 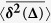 from Eqs (29), (42) and (43) (red line). Here the trace length is *t* = 10^9^ and we show the cases *α* = 1/2 (**a**) and *α* = 1/6 (**b**). Dashed lines show the asymptotics at short and long lag times. For *α* = 1/2 the transition between ballistic behaviour at short times, 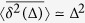, and the linear regime at long times, 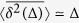, is observed. For *α* = 1/6 an additional transient regime becomes obvious due to long ranging effects of the underdamped motion. The overdamped time averaged MSD is linear with respect to Δ in both cases, 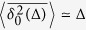. The other parameters are the same as in Fig. 1. The shape of 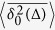 at Δ ≈ *t* is dominated by the pole in definition at which 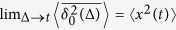, see also below in Fig. 4.

In Fig. 2 the results of numerical integration of Eqs (29), (42) and (43) for longer trace length *t* = 10^9^ are presented. While for *α* = 1/2 in panel 2a) the ballistic regime for 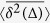 directly crosses over to the asymptotic linear behaviour, for the smaller value *α* = 1/6 the additional intermediate regime is distinct, Fig. 2b). In contrast, the overdamped values of the time averaged MSD have a linear dependence on the lag time during the whole observation time and do therefore fail to adequately describe the behaviour of the system in the case of subdiffusion, if only the anomalous diffusion exponent *α* is sufficiently small. We note that the value *α* = 1/6 characterises the subdiffusion in a granular gas with relative velocity dependent restitution coefficient, see section IVB. Also, lipid molecules in a gel phase bilayer display *α* ≈ 0.16^34^. Small *α* values, inter alia, can also be tuned for the motion of submicron beads in actin meshes^85^ or for the generic motion in glassy systems as described by the quenched trap model^86^.

#### Ultraslow underdamped scaled Brownian motion with *α* = 0

We now turn to the special case of ultraslow UDSBM governed by the Langevin equation (25) with *α* = 0,


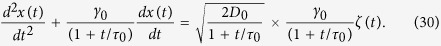


In this case the velocity correlation function attains the power law time dependence


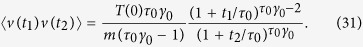


The MSD can be easily calculated from this velocity correlation function, yielding


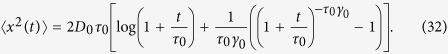


At times *t* ≪ *τ*_0_ the temperature of the system does not significantly change and the MSD behaves as if the temperature were constant, the case captured by Eq. (12). Namely, for *t* ≪ 1/*γ*_0_ the MSD has the ballistic time dependence 〈*x*^2^(*t*)〉 = (*T*_0_/*m*)*t*^2^ and at intermediate times 1/*γ*_0_ ≪ *t* ≪ *τ*_0_ normal diffusion of the form 〈*x*^2^(*t*)〉 = 2*D*_0_*t* is obtained. In the long time limit it scales logarithmically as in the case of ultraslow SBM is given by Eq. (19)^75^. The behaviour of the MSD in the ultraslow limit *α* = 0 is depicted in Fig. 1 by the black line.

The time averaged MSD for ultraslow UDSBM may also be presented as a sum of two terms according to Eq. (29). At short lag times Δ ≪ 1/*γ*_0_ the time averaged MSD scales ballistically, 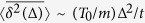. At intermediate lag times *τ*_0_ ≪ Δ ≪ *t*/(*τ*_0_*γ*_0_) the overdamped time averaged MSD given by Eq. (20) is cancelled out and the underdamped time averaged MSD has the precise linear dependence on the lag time Δ


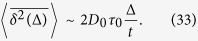


At longer lag times *t*/(*γ*_0_*τ*_0_) ≪ Δ ≪ *t* the main term *δ*_0_(Δ) ≫ Ξ(Δ) starts to dominate and the overdamped regime according to Eq. (20) is observed.

**Figure 3:**
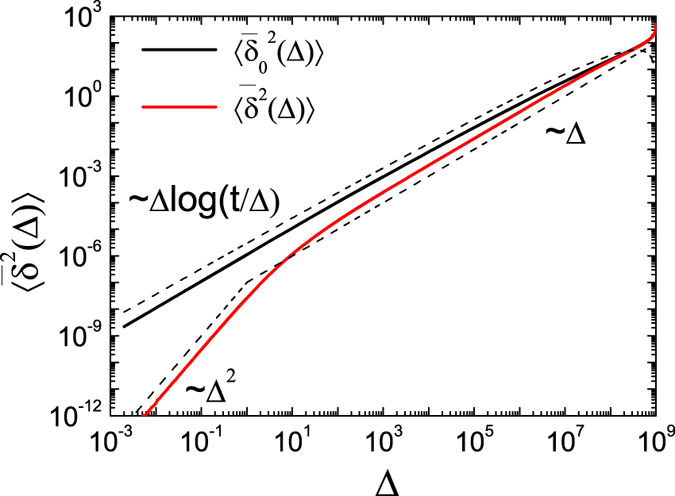
Time averaged MSD in the underdamped limit, 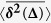 according to Eqs (29), (61) and (62) (red line), and in the overdamped limit, 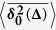 according to Eq. (61) (black line), for ultraslow UDSBM. The measurement time is *t* = 10^9^, and we chose *γ*_0_ = 1, *τ*_0_ = 30, *D*_0_ = 1, *m* = 1, and *T*_0_ = 1. For the underdamped time averaged MSD the crossover between the ballistic behaviour at short times 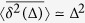 and the linear regime at long times 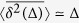 is observed. The overdamped time averaged MSD scales as 
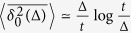
 according to Eq. (20).

This analytical result is corroborated by Fig. 3 showing the comparison between the under- and overdamped behaviours of the time averaged MSD for ultraslow UDSBM. In the underdamped case the time averaged MSD 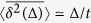 has the linear slope (33) while in the overdamped case it has the additional logarithmic correction according to Eq. (20). For the parameter values used in Fig. 3 the overdamped limit is even not visible during the entire evolution of the system. For all practical purposes, this means that the inertial corrections influence the system’s behaviour during the entire measurable time evolution. This observation accounts for the relatively small but apparent discrepancy between the granular gas simulations and the SBM description in ref. 74.

The persistent dominance of ballistic contributions for ultraslow UDSBM and thus the failure of the corresponding overdamped ultraslow SBM description is our second main result.

### Computer simulations

**Figure 4:**
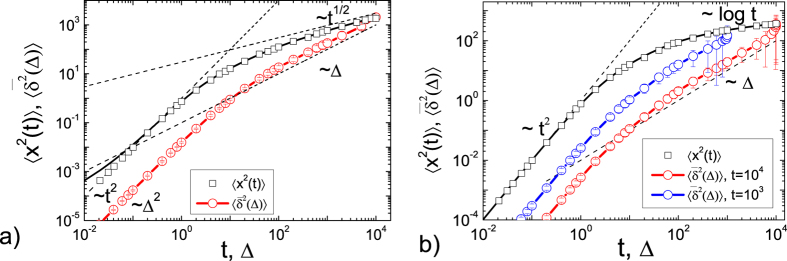
MSD 〈*x*^2^(*t*)〉 and time averaged MSD 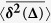 obtained from computer simulations of the corresponding finite difference analogue of the Langevin equation for *γ*_0_ = 1, *τ*_0_ = 30, *D*_0_ = 1, *m* = 1, *T*_0_ = 1. We show the cases of subdiffusion with *α* = 1/2 (panel a) and of ultraslow diffusion with *α* = 0 (panel b). The symbols depict the simulations results of the Langevin equations (25) (a) and (30) (b). The lines represent the analytical results (28) and (32), respectively.

**Figure 5:**
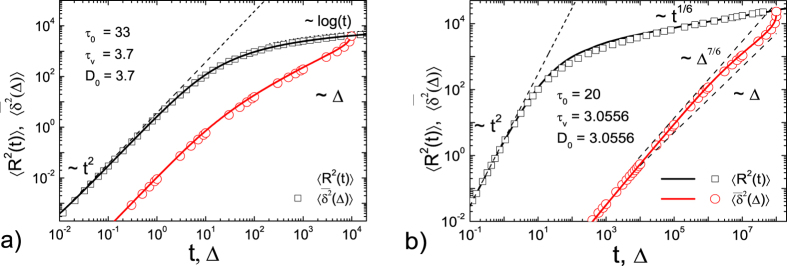
MSD 〈*x*^2^(*t*)〉 and time averaged MSD 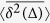 from event driven computer simulations of granular gases with constant restitution coefficient (**a**) and relative velocity dependent restitution coefficient with *α* = 1/6 (**b**). Symbols correspond to simulation results, the lines represent the analytical results of our UDSBM model, Eqs (28), (42) and (43) for panel (a), and Eqs (32), (61) and (62) for panel (b). Excellent agreement is observed.

Here we demonstrate that our analytical results for UDSBM obtained above are indeed confirmed by computer simulations of the corresponding finite-difference analogues of the Langevin equations (Fig. 4) and by event driven simulations of granular gases (Fig. 5).

#### Finite difference analogue of the Langevin equation

The finite-difference analogue of the Langevin equation may be implemented in the following way,


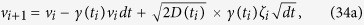



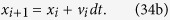


Here *dt* = *t*_*i*+1_ − *t*_*i*_ is the time step, *v*_*i*_ = *v*(*t*_*i*_) and *x*_*i*_ = *x*(*t*_*i*_) are the velocity and coordinate of a Brownian particle at the time *t*_*i*_, respectively. *ζ*_*i*_ is a random number distributed according to a standard normal distribution generated using the Box-Muller transform.

The comparison of the simulations of the finite difference analogue of the Langevin equation with the theory for *α* = 1/2 and *α* = 0 are shown in Fig. 4a,b, respectively. The symbols denote the results of the computer simulation and the lines represent the analytical results. The simulations results are in excellent agreement with our analytical results. At short times both MSD and time averaged MSD exhibit the expected ballistic behaviour. At long times the MSD scales as 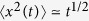 for *α* = 1/2 and as 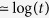 for *α* = 0. The time averaged MSD scales linearly at long lag times in both cases. For the ultraslow case with *α* = 0 this fact underlines the remarkable and non-negligible persistence of the ballistic effects.

#### Event driven simulations of granular gases

In the event driven Molecular Dynamics simulations shown in Fig. 5 we study a gas of hard sphere granular particles of unit mass and radius, colliding respectively with constant and viscoelastic restitution coefficients. Our simulations code is based on the algorithm suggested in ref. 87. The particles move freely between pairwise collisions, during the collisions the particle velocities are updated according to certain collisional rules. The duration time of the collisions is equal to zero, that is, the velocities of particles are updated instantaneously. Other details of the event driven simulations are provided in ref. 74. As a three dimensional granular gas is simulated, in order to compare with our theory all results for the moments should be divided by the factor 3.

At short times both the MSD and the time averaged MSD show a ballistic (lag) time dependence. At long times the ensemble averaged MSD 〈*x*^2^(*t*)〉 scales according as 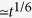 for *α* = 1/6 and as 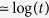 for *α* = 0 (see the two panels of Fig. 5). The time averaged MSD 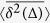 scales linearly for the granular gas with constant restitution coefficient, as in the case of ultraslow UDSBM (Fig. 5a). The time averaged MSD shows a distinct crossover behaviour for a granular gas with velocity dependent restitution coefficient, as well as SBM with *α* = 1/6 (Fig. 5b). These observations demonstrate that both qualitatively and quantitatively the behaviour of granular gases with constant and velocity dependent restitution coefficients is fully captured by our UDSBM model. The intermediate time deviations observed in our earlier study^74^ are thus remedied by the inclusion of explicit long-ranging underdamped effects. The full agreement of the UDSBM model with the granular gas dynamics is our third main result and thus provides an interesting and easy to analytically implement model for granular gas dynamics in the homogeneous cooling state for both constant and velocity dependent restitution coefficients.

## Discussion

We established and studied UDSBM in terms of an underdamped Langevin equation with time dependent temperature and consequently time dependent diffusion and damping coefficients. We derived the MSD and its time averaged analogue. As the main findings we demonstrated that the overdamped analogue of UDSBM, the well known SBM process, fails to adequately capture the behaviour of an UDSBM particle even in the long time limit. Instead for pronounced subdiffusion there exists a persistent intermediate regime for the time averaged MSD which leads to deviations from the overdamped solution. In the ultraslow case these corrections persist practically forever. For both cases with *α* > 0 and *α* = 0 the corrections to the behaviour captured by the overdamped SBM Langevin equation were corroborated by simulations of the finite difference UDSBM Langevin equation and event driven Molecular Dynamics simulations of cooling granular gases. In other words, effects of inertia play a significant role even at relatively long times and neglecting the inertial term in the Langevin equation may lead to an incorrect description of the physical properties of the system. Given the high accuracy achieved by modern experimental tools tracing diffusing particles in complex environments or the possibility to run simulations over large time windows a proper description in terms of the full underdamped dynamics is thus highly important. This fact was demonstrated here by comparison to simulations of granular gases with time dependent temperature (kinetic energy).

SBM can readily be extended to include an inertial term, as shown here. It can therefore be directly compared to fractional Langevin equation motion. These two families of anomalous stochastic processes are in some sense opposites: fractional Langevin equation motion has stationary increments but is highly non-Markovian, whereas UDSBM is Markovian yet fully non-stationary. For fractional Langevin equation motion effects of ballistic contributions were observed for the fractional Langevin equation, leading to oscillations in the velocity correlations^60^,^88^. Moreover, transient ageing and weak ergodicity breaking were observed in these systems^27^,^61^,^62^. However, these effects decay relatively quickly. For UDSBM, in particular for small or vanishing values of the anomalous diffusion exponent *α*, these ballistic correlations turn out to be very persistent and were shown here to be necessary to explain the full behaviour of physical systems such as granular gases. How generic such features are for other non-stationary anomalous diffusion processes such as heterogeneous diffusion processes with position dependent diffusion coefficient or continuous time random walks will therefore be an important question.

Our results demonstrate that good care is needed for the physically correct description of anomalous diffusion processes: the naive assumption of the equivalence of the long time behaviour and the overdamped description is not always correct and may lead to false conclusions.

## Methods

### Underdamped scaled brownian motion with *α* > 0

The solution of the Langevin equation (25) has the form


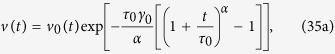



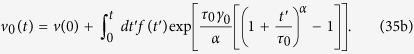


Here 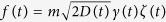, the right hand side of Eq. (25). The velocity correlation function then yields as (*t*_2_ > *t*_1_)


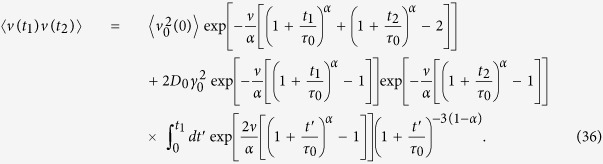


Here *ν* = *τ*_0_*γ*_0_. Changing the variables in the integral,


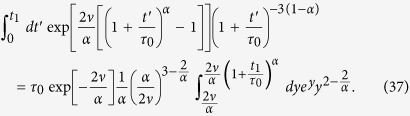


Taking into account that *e*^*x*^ is a fast growing function, we approximate the integral in the following way,


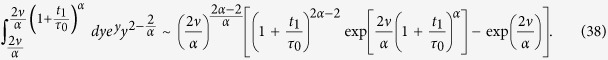


From the ensuing velocity correlation function with 
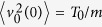
 we arrive at Eq. (27).

The time averaged MSD is defined as


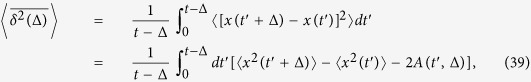


where


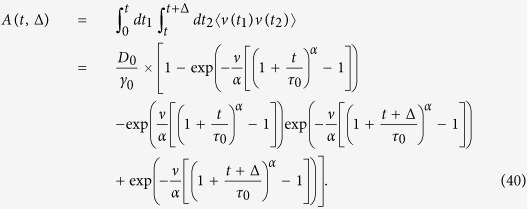


The integrand in Eq. (39) attains with the velocity correlation function (36) the following form


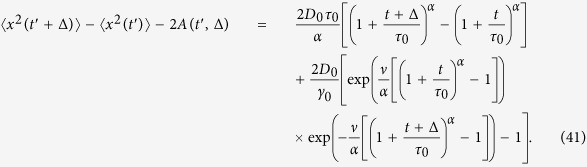


The time averaged MSD may be presented as the sum of two terms according to Eq. (29). The first term corresponds to the time averaged MSD in the overdamped (SBM) limit,


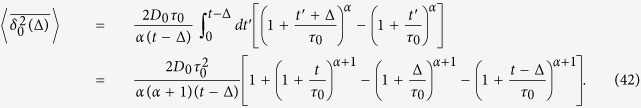


The second part in the time averaged MSD reads


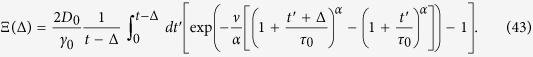


#### Short lag times: *γ*_0_,Δ ≪  *t* ≪ *τ*_0_

From Eqs (42) and (43) we find that 
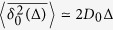
 and 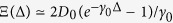, their combination yielding the total time averaged MSD


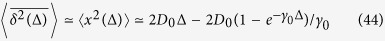


#### Long lag times: *τ*_0_ ≪ Δ ≪  *t*

For the 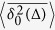 term we obtain Eq. (17) from the main text, namely


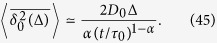


Let us now consider the additional contribution coming from Eq. (43). We can rewrite this equation via change of variables,


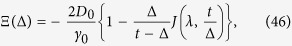


where the functions are defined as


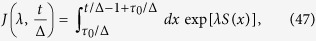


as well as


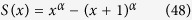


and


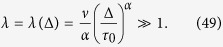


We have to consider superdiffusive and subdiffusive situations separately.

#### Superdiffusion, *α* > 1

For superdiffusion the maximum of *S*(*x*) is achieved at the lower limit of the integral (47), namely


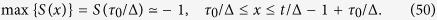


We estimate the integral (47) with the method of steepest descent,


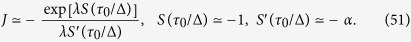


Therefore, we find that *J* gives an exponentially small contribution to Ξ(Δ) and


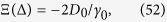


that is


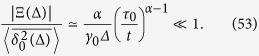


Thus, the overdamped result for the time averaged MSD provides the correct result in the superdiffusive case.

#### Subdiffusion, 0 < *α* < 1

In the subdiffusive case the maximum of *S*(*x*) is achieved at the upper limit of the integral (47),


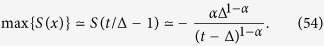


For longer lag times, such that


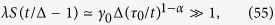


that is 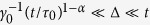, the contribution of *J* is again exponentially small and we have—similarly to the superdiffusive case—that


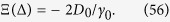


Thus, due to Eq. (55) we see that


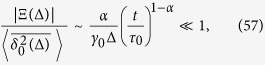


and the time averaged MSD corresponds to the overdamped approximation. In contrast, for shorter lag times,


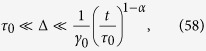


the method of the steepest descent is not valid. We may roughly estimate the lower bound of |Ξ(Δ)| as


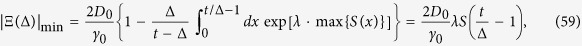


and thus


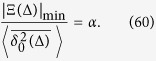


This estimate shows that in the domain of variables (58) the contributions to the time averaged MSD stemming from the terms 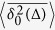 and Ξ(Δ) are of comparable magnitude, and thus inertial effects cannot be neglected in the consideration.

### Ultraslow underdamped scaled brownian motion with *α* = 0

Ultraslow UDSBM corresponds to the case *α* = 0 in which the velocity correlation function (31) and the MSD (32) may be obtained from the results of the previous section in the limit *α* → 0 taking into account that 
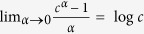
.

The first term of the time averaged MSD corresponds to the time averaged MSD for ultraslow SBM,


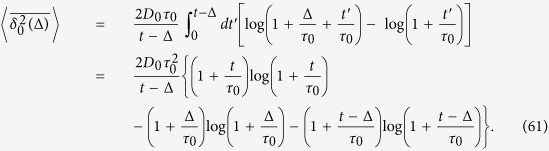


The second term may be derived analogously to the previous section,


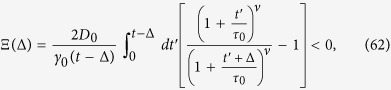


where we took into account that *ν* = *τ*_0_*γ*_0_ ≫ 1. In what follows we consider separately the limits of short and long lag times.

#### Short lag times, Δ ≪ *t* ≪ *τ*_0_

From Eqs (61) and (62) we find


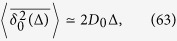


and


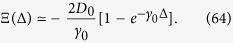


By combining expressions (63) and (64) we get the time averaged MSD


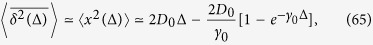


as expected for short lag times, see Eq. (13) of the main text. Note the approximate sign in Eq. (65) because it is valid up to terms that are smaller by the factor *t*/*τ*_0_.

#### Long lag times, *τ*_0_ ≪ Δ ≪ *t*

The contribution given by relation (61) can be calculated directly,


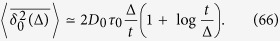


Changing variables in the integrand of Eq. (62) we rewrite it as


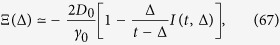


where we define


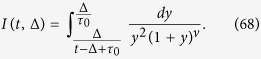


Since the integrand is decaying fast at *y* → ∞, we can safely replace the upper limit of the integral by ∞. Moreover we can neglect the term *τ*_0_ at the lower integration limit. Then we integrate by parts twice in order to extract the main terms such that


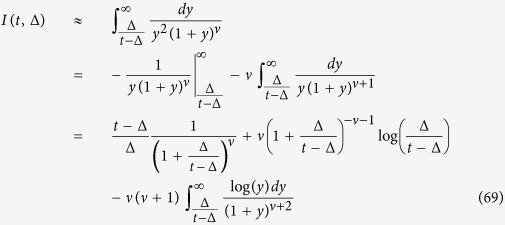


The integrand in the last term of the right hand side has an integrable divergence at zero, thus we can safely put the lower limit to zero and use^89^


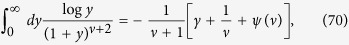


where *γ* = 0.5772… is Euler’s constant and 
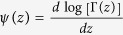
 is the digamma function. After plugging (70) into (69) and then (69) into (67) we get


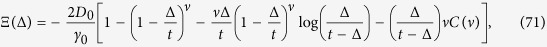


where the following definition is introduced


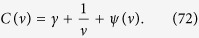


Equation (71) exhibits two different behaviours in the long time limit considered here. Thus, for *τ*_0_ ≪ Δ ≪ *t*/*ν* we find


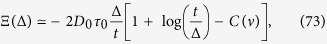


and by combining (68) and (75) we observe the cancellation of the main terms in 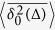 and Ξ(Δ), resulting for the time averaged MSD in


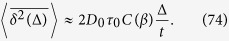


For longer lag times *τ*_0_ ≪ *t*/*ν* ≪ Δ ≪ *t* Eq. (71) yields


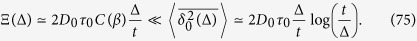


Thus, in this case the main term of 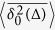 is not cancelled out, and the overdamped regime (19) of the main text is observed.

## Additional Information

**How to cite this article**: Bodrova, A. S. *et al*. Underdamped scaled Brownian motion: (non-)existence of the overdamped limit in anomalous diffusion. *Sci. Rep*. **6**, 30520; doi: 10.1038/srep30520 (2016).

## References

[b1] Fick A. Über Diffusion. Ann. Phys. (Leipzig) 170, 59–86 (1855).

[b2] Einstein A. Über die von der molekularkinetischen Theorie der Wärme geforderte Bewegung von in ruhenden Flüssigkeiten suspendierten Teilchen. Ann. Phys. (Leipzig) 17, 549–560 (1905).

[b3] von Smoluchowski M. Zur kinetischen Theorie der Brownschen Molecularbewegung und der Suspensionen, Ann. Phys. (Leipzig) 21, 756–780 (1906).

[b4] Richardson L. F. In General Systems: Yearbook of the Society for General Systems Research, vol. VI (1961).

[b5] Bouchaud J.-P. & Georges A. Anomalous diffusion in disordered media: statistical mechanisms, models and physical applications. Phys. Rep. 195, 127–293 (1990).

[b6] Metzler R. & Klafter J. The random walk’s guide to anomalous diffusion: a fractional dynamics approach. Phys. Rep. 339, 1–77 (2000).

[b7] Sokolov I. M. Models of anomalous diffusion in crowded environments. Soft Matter 8, 9043–9052 (2012).

[b8] Metzler R., Jeon J.-H., Cherstvy A. G. & Barkai E. Anomalous diffusion models and their properties: non-stationarity, non-ergodicity, and ageing at the centenary of single particle tracking. Phys. Chem. Chem. Phys. 16, 24128–24164 (2014). 25297814 10.1039/c4cp03465a

[b9] Scher H. & Montroll E. W. Anomalous transit-time dispersion in amorphous solids. Phys. Rev. B 12, 2455–2477 (1975).

[b10] Berkowitz B., Cortis A., Dentz M. & Scher H. Modeling non-Fickian transport in geological formations as a continuous time random walk. Rev. Geophys. 44, RG2003 (2006).

[b11] Young W., Pumir A. & Pomeau Y. Diffusion of tracer in convection rolls. Phys. Fluids A1, 462 (1989).

[b12] Lizana L., Ambjörnsson T., Taloni A., Barkai E. & Lomholt M. Foundation of fractional Langevin equation: Harmonization of a many-body problem. Phys. Rev. E 81, 051118 (2010). 10.1103/PhysRevE.81.051118 20866196

[b13] Solomon T. H., Weeks E. R. & Swinney H. L. Observation of anomalous diffusion and Lévy flights in a two-dimensional rotating flow. Phys. Rev. Lett. 71, 3975–3978 (1993). 10055122 10.1103/PhysRevLett.71.3975

[b14] Ariel G. . Swarming bacteria migrate by Lévy Walk. Nature Comm. 6, 8396 (2015). 10.1038/ncomms9396 PMC4598630 26403719

[b15] Humphries N. E. . Foraging success of biological Lévy flights recorded *in situ*. Proc. Natl Acad. Sci. USA 109 7169–7174 (2012). 22529349 10.1073/pnas.1121201109 PMC3358854

[b16] Brockmann D. Following the Money. Phys. World, 2, 31–34 (2010).

[b17] Meroz Y. & Sokolov I. M. A toolbox for determining subdiffusive mechanisms. Phys. Rep. 573, 1–29 (2015).

[b18] Barkai E., Garini Y. & Metzler R. Strange kinetics of single molecules in living cells. Phys. Today 65, 29–35 (2012).

[b19] Höfling F. & Franosch T. Anomalous transport in the crowded world of biological cells. Rep. Prog. Phys. 76, 046602 (2013). 23481518 10.1088/0034-4885/76/4/046602

[b20] Golding I. & Cox E. C. Physical Nature of Bacterial Cytoplasm. Phys. Rev. Lett. 96, 098102 (2006). 16606319 10.1103/PhysRevLett.96.098102

[b21] Bronstein I. . Transient Anomalous Diffusion of Telomeres in the Nucleus of Mammalian Cells. Phys. Rev. Lett. 103, 018102 (2009). 19659180 10.1103/PhysRevLett.103.018102

[b22] Jeon J.-H. . *In Vivo* Anomalous Diffusion and Weak Ergodicity Breaking of Lipid Granules. Phys. Rev. Lett. 106, 048103 (2011). 21405366 10.1103/PhysRevLett.106.048103

[b23] Tabei S. M. A. . Intracellular transport of insulin granules is a subordinated random walk. Proc. Natl. Acad. Sci. USA 110, 4911–4916 (2013). 23479621 10.1073/pnas.1221962110 PMC3612641

[b24] Di Rienzo C., Piazza V., Gratton E., Beltram F. & Cardarelli F. Probing short-range protein brownian motion in the cytoplasm of living cells. Nature Comm. 5, 5891 (2014). 10.1038/ncomms6891 PMC4281647 25532887

[b25] Szymanski J. & Weiss M. Elucidating the Origin of Anomalous Diffusion in Crowded Fluids. Phys. Rev. Lett. 103, 038102 (2009). 19659323 10.1103/PhysRevLett.103.038102

[b26] Pan W. . Viscoelasticity in Homogeneous Protein Solutions. Phys. Rev. Lett. 102, 058101 (2009). 19257559 10.1103/PhysRevLett.102.058101

[b27] Jeon J.-H., Leijnse N., Oddershede L. B. & Metzler R. Anomalous diffusion and power-law relaxation of the time averaged mean squared displacement in worm-like micellar solutions. New J. Phys. 15, 045011 (2013).

[b28] Sentjabrskaja T. . Anomalous dynamics of intruders in a crowded environment of mobile obstacles. Nature Comm. 7, 11133 (2016). 10.1038/ncomms11133 PMC4822008 27041068

[b29] Caspi A., Granek R. & Elbaum M. Enhanced Diffusion in Active Intracellular Transport. Phys. Rev. Lett. 85, 5655–5658 (2000). 11136070 10.1103/PhysRevLett.85.5655

[b30] Robert D., Nguyen T. H., Gallet F. & Wilhelm C. *In Vivo* Determination of Fluctuating Forces during Endosome Trafficking Using a Combination of Active and Passive Microrheology. PLoS ONE 5, e10046 (2010). 20386607 10.1371/journal.pone.0010046 PMC2850365

[b31] Reverey J. F., Jeon J.-H., Leippe M., Metzler R. & Selhuber-Unkel C. Superdiffusion dominates intracellular particle motion in the supercrowded cytoplasm of pathogenic *Acanthamoeba castellanii*. Sci. Rep. 5, 11690 (2015). 26123798 10.1038/srep11690 PMC5155589

[b32] Hu X. . The dynamics of single protein molecules is non-equilibrium and self-similar over thirteen decades in time. Nature Phys. 12, 171–174 (2016).

[b33] Kneller G. R., Baczynski K. & Pasenkiewicz-Gierula M. Communication: Consistent picture of lateral subdiffusion in lipid bilayers: Molecular dynamics simulation and exact results. J. Chem. Phys. 135, 141105 (2011). 22010688 10.1063/1.3651800

[b34] Jeon J.-H., Martinez-Seara Monne H., Javanainen M. & Metzler R. Anomalous Diffusion of Phospholipids and Cholesterols in a Lipid Bilayer and its Origins. Phys. Rev. Lett. 109, 188103 (2012). 23215336 10.1103/PhysRevLett.109.188103

[b35] Javanainen M. . Anomalous and normal diffusion of proteins and lipids in crowded lipid membranes. Faraday Disc. 161, 397–417 (2013). 10.1039/c2fd20085f 23805752

[b36] Jeon J.-H., Javanainen M., Martinez-Seara H., Metzler R. & Vattulainen I. Phys. Rev. X 6, 021006 (2016).

[b37] Metzler R., Jeon J.-H. & Cherstvy A. G. Non-Brownian diffusion in lipid membranes: Experiments and simulations. Biophys. Biochem. Acta, doi: 10.1016/j.bbamem.2016.01.022 (2016). 26826272

[b38] Sinai Ya. G. The Limiting Behavior of a One-Dimensional Random Walk in a Random Medium. Theory Prob. Appl. 27, 256–268 (1982).

[b39] Godec A., Chechkin A. V., Barkai E., Kantz H. & Metzler R. Localization and universal fluctuations in ultraslow diffusion processes. J. Phys. A 47, 492002 (2014).

[b40] Dräger J. & Klafter J. Strong Anomaly in Diffusion Generated by Iterated Maps. Phys. Rev. Lett. 84, 5998–6001 (2000). 10991108 10.1103/PhysRevLett.84.5998

[b41] Sperl M. Nearly logarithmic decay in the colloidal hard-sphere system. Phys. Rev. E 71, 060401 (2005). 10.1103/PhysRevE.71.060401 16089713

[b42] Cassi D. & Regina S. Random Walks on Bundled Structures. Phys. Rev. Lett. 76, 2914–2917 (1996). 10060823 10.1103/PhysRevLett.76.2914

[b43] Sanders L. P . Severe slowing-down and universality of the dynamics in disordered interacting many-body systems: ageing and ultraslow diffusion. New J. Phys. 16, 113050 (2014).

[b44] Brilliantov N. V. & Pöschel T. Kinetic Theory of Granular Gases, Oxford University Press, Oxford (2004).

[b45] Klafter J., Blumen A. & Shlesinger M. F. Stochastic pathway to anomalous diffusion. Phys. Rev. A 35, 3081–2085 (1987). 10.1103/physreva.35.3081 9898509

[b46] Goychuk I. Viscoelastic Subdiffusion: Generalized Langevin Equation Approach. Adv. Chem. Phys. 150, 187 (2012).

[b47] Cherstvy A. G., Chechkin A. V. & Metzler R. Anomalous diffusion and ergodicity breaking in heterogeneous diffusion processes. New J. Phys. 15, 083039 (2013).

[b48] Massignan P. . Nonergodic Subdiffusion from Brownian Motion in an Inhomogeneous Medium. Phys. Rev. Lett. 112, 150603 (2014). 24785018 10.1103/PhysRevLett.112.150603

[b49] Havlin S. & Weiss G. H. A New Class of Long-Tailed Pausing Time Densities for the CTRW. J. Stat. Phys. 58, 1267–1273 (1990).

[b50] Cherstvy A. G. & Metzler R. Population splitting, trapping, and non-ergodicity in heterogeneous diffusion processes. Phys. Chem. Chem. Phys. 15, 20220–20235 (2013). 24162164 10.1039/c3cp53056f

[b51] Langevin P. On the Theory of Brownian Motion. C. R. Acad. Sci. (Paris) 146, 530–533 (1908).

[b52] van Kampen N. G. Stochastic processes in physics and chemistry (North Holland, Amsterdam, 1981).

[b53] Risken H. The Fokker-Planck equation (Springer, Heidelberg, 1989).

[b54] Lim S. C. & Muniandy S. V. Self-similar Gaussian processes for modeling anomalous diffusion. Phys. Rev. E 66, 021114 (2002). 10.1103/PhysRevE.66.021114 12241157

[b55] Jeon J.-H., Chechkin A. V. & Metzler R. Scaled Brownian motion: a paradoxical process with a time dependent diffusivity for the description of anomalous diffusion. Phys. Chem. Chem. Phys. 16, 15811–15817 (2014). 24968336 10.1039/c4cp02019g

[b56] Thiel F. & Sokolov I. M. Scaled Brownian motion as a mean-field model for continuous-time random walks. Phys. Rev. E 89, 012115 (2014). 10.1103/PhysRevE.89.012115 24580180

[b57] Safdari H. . Quantifying the non-ergodicity of scaled Brownian motion. J. Phys. A 48, 375002 (2015).

[b58] Safdari H., Chechkin A. V., Jafari G. R. & Metzler R. Aging Scaled Brownian Motion. Phys. Rev. E 91, 042107 (2015). 10.1103/PhysRevE.91.042107 25974439

[b59] Hänggi P. Correlation functions and master equations of generalized (non-Markovian) Langevin equations. Z. Physik B 31, 407–416 (1978).

[b60] Burov S. & Barkai E. Critical Exponent of the Fractional Langevin Equation. Phys. Rev. Lett. 100, 070601 (2008). 18352535 10.1103/PhysRevLett.100.070601

[b61] Jeon J.-H. & Metzler R. Inequivalence of time and ensemble averages in ergodic systems: exponential versus power-law relaxation in confinement. Phys. Rev. E 85, 021147 (2012). 10.1103/PhysRevE.85.021147 22463192

[b62] Kursawe J., Schulz J. H. P. & Metzler R. Transient ageing in fractional Brownian and Langevin equation motion. Phys. Rev. E 88, 062124 (2013). 10.1103/PhysRevE.88.062124 24483403

[b63] Batchelor G. K. Diffusion in a field of homogeneous turbulence. Math. Proc. Cambridge Philos. Soc. 48, 345–362 (1952).

[b64] Novikov D. S., Jensen J. H., Helpern J. A. & Fieremans E. Revealing mesoscopic structural universality with diffusion. Proc. Natl. Acad. Sci. USA 111, 5088–5093 (2014). 24706873 10.1073/pnas.1316944111 PMC3986157

[b65] Feder T. J., Brust-Mascher I., Slattery J. P., Baird B. & Webb W. W. Constrained diffusion or immobile fraction on cell surfaces: a new interpretation. Biophys. J. 70, 2767–2773 (1996). 8744314 10.1016/S0006-3495(96)79846-6 PMC1225256

[b66] Sen P. N. Time-dependent diffusion coefficient as a probe of geometry. Conc. Magnetic Reson. 23A, 1–21 (2004).

[b67] Saxton M. J. Anomalous subdiffusion in fluorescence photobleaching recovery: a Monte Carlo study. Biophys. J. 81, 2226–2240 (2001). 11566793 10.1016/S0006-3495(01)75870-5 PMC1301694

[b68] Schwille P., Haupts U., Maiti S. & Webb W. W. Molecular dynamics in living cells observed by fluorescence correlation spectroscopy with one- and two-photon excitation. Biophys. J. 77, 2251–2265 (1999). 10512844 10.1016/S0006-3495(99)77065-7 PMC1300505

[b69] Guigas G., Kalla C. & Weiss M. The degree of macromolecular crowding in the cytoplasm and nucleoplasm of mammalian cells is conserved. FEBS Lett. 581, 5094–5098 (2007). 17923125 10.1016/j.febslet.2007.09.054

[b70] Molini A., Talkner P., Katul G. G. & Porporato A. First passage time statistics of Brownian motion with purely time dependent drift and diffusion. Physica A 390, 1841–1852 (2011).

[b71] De Walle D. & Rango A. Principles of Snow Hydrology, Cambridge University Press (Cambridge, UK, 2008).

[b72] Poeschel T. & Luding S. edited by Granular Gases, Lecture Notes in Physics Vol. 564 (Springer, Berlin, 2001).

[b73] Poeschel T. & Brilliantov N. V. edited by Granular Gas Dynamics Lecture Notes in Physics. Vol. 624 (Springer, Berlin, 2003).

[b74] Bodrova A. S., Chechkin A. V., Cherstvy A. G. & Metzler R. Quantifying non-ergodic dynamics of force-free granular gases. Phys. Chem. Chem. Phys. 17, 21791–21798 (2015). 26252559 10.1039/c5cp02824h

[b75] Bodrova A. S., Chechkin A. V., Cherstvy A. G. & Metzler R. Ultraslow scaled Brownian motion. New J. Phys. 17, 063038 (2015).

[b76] Klimontovich Yu. L. Statistical physics (Harwood Academic publishers, Chur, 1986).

[b77] Bodrova A. S. & Brilliantov N. V. Self-diffusion in granular gases: an impact of particles roughness Granular Matter 14, 85–90 (2012).

[b78] Haff P. K. Grain flow as a fluid-mechanical phenomenon. J. Fluid Mech. 134, 401–430 (1983).

[b79] Brilliantov N. V. & Pöschel T. Self-diffusion in granular gases. Phys. Rev. E 61, 1716–1721 (2000). 10.1103/physreve.61.1716 11046456

[b80] Brey J. J., Ruiz-Montero M. J., Cubero D. & Garcia-Rojo R. Self-diffusion in freely evolving granular gases. Phys. of Fluids 12, 876–883 (2000).

[b81] Dufty J. W., Brey J. J. & Lutsko J. Diffusion in a granular fluid. I. Theory. Phys. Rev. E 65, 051303 (2002). 10.1103/PhysRevE.65.051303 12059547

[b82] Lutsko J., Brey J. J. & Dufty J. W. Diffusion in a granular fluid. II. Simulation. Theory. Phys. Rev. E, 65, 051304 (2002). 10.1103/PhysRevE.65.051304 12059548

[b83] Bodrova A. S., Dubey A. K., Puri S. & Brilliantov N. V. Intermediate Regimes in Granular Brownian Motion: Superdiffusion and Subdiffusion. Phys. Rev. Lett. 109, 178001 (2012). 23215224 10.1103/PhysRevLett.109.178001

[b84] Brey J. J. & Casado J. Generalized Langevin Equations with Time-Dependent Temperature. J. Stat. Phys. 61, 713–722 (1990).

[b85] Wong I. Y. . Anomalous diffusion probes microstructure dynamics of entangled F-actin networks. Phys. Rev. Lett. 92, 178101 (2004). 15169197 10.1103/PhysRevLett.92.178101

[b86] Monthus C. & Bouchaud J. P. Models of traps and glass phenomenology. J. Phys. A 29, 3847–3869 (1996).

[b87] Pöschel T. & Schwager T. Computational Granular Dynamics (Springer, Berlin, 2005).

[b88] Burov S. & Barkai E. Fractional Langevin equation: Overdamped, underdamped, and critical behaviors. Phys. Rev. E 78, 031112 (2008). 10.1103/PhysRevE.78.031112 18850998

[b89] Prudnikov A. P., Brychkov Yu. A. & Marichev O. I. Integrals & Series, Volume 1: Elementary functions (Gordon & Breach, New York, 1998).

